# Development and validation of explainable machine-learning models for carotid atherosclerosis early screening

**DOI:** 10.1186/s12967-023-04093-8

**Published:** 2023-05-29

**Authors:** Ke Yun, Tao He, Shi Zhen, Meihui Quan, Xiaotao Yang, Dongliang Man, Shuang Zhang, Wei Wang, Xiaoxu Han

**Affiliations:** 1 National Clinical Research Center for Laboratory Medicine, The First Affiliated Hospital of China Medical University, Shenyang, Liaoning Province China; 2 Department of Laboratory Medicine, The First Affiliated Hospital of China Medical University, Shenyang, Liaoning Province China; 3 Neusoft Research Institute, Neusoft Corporation, Shenyang, Liaoning Province China; 4 Department of Software Engineering, Northeastern University, Shenyang, Liaoning Province China; 5 Department of Physical Examination Center, The First Affiliated Hospital of China Medical University, Shenyang, Liaoning Province China; 6 Laboratory Medicine Innovation Unit, Chinese Academy of Medical Sciences, Shenyang, Liaoning Province China; 7 NHC Key Laboratory of AIDS Immunology (China Medical University), The First Affiliated Hospital of China Medical University, Shenyang, Liaoning Province China

**Keywords:** Machine learning, Carotid atherosclerosis, Explainable model

## Abstract

**Background:**

Carotid atherosclerosis (CAS), an important factor in the development of stroke, is a major public health concern. The aim of this study was to establish and validate machine learning (ML) models for early screening of CAS using routine health check-up indicators in northeast China.

**Methods:**

A total of 69,601 health check-up records from the health examination center of the First Hospital of China Medical University (Shenyang, China) were collected between 2018 and 2019. For the 2019 records, 80% were assigned to the training set and 20% to the testing set. The 2018 records were used as the external validation dataset. Ten ML algorithms, including decision tree (DT), K-nearest neighbors (KNN), logistic regression (LR), naive Bayes (NB), random forest (RF), multiplayer perceptron (MLP), extreme gradient boosting machine (XGB), gradient boosting decision tree (GBDT), linear support vector machine (SVM-linear), and non-linear support vector machine (SVM-nonlinear), were used to construct CAS screening models. The area under the receiver operating characteristic curve (auROC) and precision-recall curve (auPR) were used as measures of model performance. The SHapley Additive exPlanations (SHAP) method was used to demonstrate the interpretability of the optimal model.

**Results:**

A total of 6315 records of patients undergoing carotid ultrasonography were collected; of these, 1632, 407, and 1141 patients were diagnosed with CAS in the training, internal validation, and external validation datasets, respectively. The GBDT model achieved the highest performance metrics with auROC of 0.860 (95% CI 0.839–0.880) in the internal validation dataset and 0.851 (95% CI 0.837–0.863) in the external validation dataset. Individuals with diabetes or those over 65 years of age showed low negative predictive value. In the interpretability analysis, age was the most important factor influencing the performance of the GBDT model, followed by sex and non-high-density lipoprotein cholesterol.

**Conclusions:**

The ML models developed could provide good performance for CAS identification using routine health check-up indicators and could hopefully be applied in scenarios without ethnic and geographic heterogeneity for CAS prevention.

## Introduction

Carotid atherosclerosis (CAS) is a vital risk factor for cardiovascular and cerebral events. It is characterized by pathological thickening of the common or internal carotid intima, and because of the increased risk of ischemic stroke, coronary events, and blood flow restriction, it is a non-negligible disease burden in society worldwide [1, 2]. A recent study showed that increased carotid intima-media thickness (IMT) is projected to occur in adults aged 30–79 worldwide at a prevalence of 27.62%, carotid plaque at a prevalence of 21.13%, and carotid stenosis at a prevalence of 1.50% [3]. In China, about 31% of the general population and 39% of people aged 60 to 69 have carotid plaques, respectively [4]. CAS identification is a prerequisite for early detection and intervention in cardiovascular and cerebrovascular events, such as stroke [4, 5].

Ultrasonography is widely used to measure carotid luminal stenosis and identify patients with carotid artery atherosclerosis [6]. However, a high proportion of patients have a delayed diagnosis of CAS. The reasons for the delay might be attributed to the following: (1) CAS is usually asymptomatic, unless the patient has experienced a symptomatic ischemic stroke, transient ischemic attack, or amaurosis fugax [3]. (2) The accuracy of the routine ultrasound examination varies greatly due to operator manipulation experience, hemodynamics, and other factors. (3) Ultrasonography is generally not used for routine health checkups, especially in economically underdeveloped areas, due to its high cost [7]. Recently, with the rapid development of artificial intelligence, machine learning (ML) algorithms have overcome the limitations of the application scope of traditional statistical models and have been successfully applied in medical scenarios for its great potential to improve the accuracy and efficiency of health outcome identification from electronic health record (EHR) datasets [8], such as screening high-risk individuals for COVID-19 [9] and patients with diabetes [10]. It has also been used in CAS diagnosis [11–13]. However, the models reported have several shortcomings. First, there has been no evaluation of common ML algorithms with demonstrated performance, such as extreme gradient boosting (XGB) and gradient boosting decision trees (GBDT), with good adaptability to tabular data [14]. In addition, the previously reported models used too many uncommon physical examination indicators, which greatly limited the ease of use of the models [12]. Furthermore, external validation, calibration, and interpretability analyses of established models have not been reported, especially the sensitivity and specificity of various ML models among different high-risk subgroups of CAS. The aim of this study was to develop and validate ML models for CAS classification using routine health check-up indicators and interpret the outputs of the optimal ML model using the SHapley Additive exPlanations (SHAP) method.

## Methods

### Data collection and participant selection

The transparent reporting of a multivariable prediction model for individual prognosis or diagnosis (TRIPOD) was followed when conducting this study [15]. The health examination center of the First Affiliated Hospital of China Medical University (Shenyang, China) provided us with health check-up medical records between 2018 and 2019 in the form of excel sheets. Individuals who participated in the physical examination were mainly employees of various organizations, new recruits, and individuals who voluntarily attended health check-ups. All participants are local residents of Shenyang, China. The training set contains 80% of the 2019 health check-up data and the internal validation dataset consists of the remaining 20%. The 2018 dataset served as the external validation set. The inclusion criteria were as follows: (1) aged ≥ 18 years, (2) underwent carotid ultrasound examination, and (3) had undergone routine biochemistry blood testing, including liver function, renal function, serum lipid, and fasting serum glucose (FSG). The following were excluded: (1) age < 18 years, (2) lack of carotid ultrasound, and (3) lack of biochemistry testing.

### Variables identified

Variables in the collected datasets included demographic characteristics, clinical variables, and laboratory indices. From the 70 health check-up items, 24 demographic and biochemical candidate parameters were selected for CAS model construction according to the study design and the clinician’s advice. The selected 24 variables were: demographic characteristics (six variables), including age, sex, body mass index (BMI), waist circumference, height, and body weight; clinical characteristics (two variables), including diastolic blood pressure (DBP) and systolic blood pressure (SBP); biochemical characteristics (16 variables), including FSG, total cholesterol (TC), triglyceride (TG), high-density lipoprotein cholesterol (HDL-C), low-density lipoprotein cholesterol (LDL-C), non-high-density lipoprotein cholesterol (non-HDL-C), alkaline phosphatase (ALP), gamma-glutamyl transpeptidase (GGT), aspartate aminotransferase (AST), alanine transaminase (ALT), total protein (TP), total bilirubin (TBIL), albumin (ALB), blood urea nitrogen (BUN), creatinine (Cr), and uric acid (UA). An automatic biochemical analyzer was used to test laboratory indicators (Cobas 8000 c701 module; Roche Diagnostics, Mannheim, Germany).

### Outcome definition and assessment

Bilateral carotid IMT maxima were used as indicators to assess the degree of carotid arteriosclerosis. According to the diagnostic criteria from the textbook of diagnostic ultrasound (3rd edition) [16], the normal IMT of the carotid artery was defined as < 1.0 mm; carotid artery atherosclerosis was defined as localized thickening of the intima (1.0 mm ≤ IMT < 1.5 mm); and carotid artery plaque was defined as an IMT of 1.5 mm or greater, or at least 0.5 mm greater than the surrounding normal IMT value, or more than 50% greater than the surrounding normal IMT value, and local changes to the structure protruding to the lumen. Subsequently, increased IMT, carotid plaque, and carotid stenosis were classified into the CAS group, and other cases were classified into the control group. CAS was diagnosed by two independent clinicians by examining left and right carotid artery ultrasound reports. In cases of disagreement, consensus was reached through discussion and consultation.

### Feature selection, model construction, and evaluation

To ensure better model discrimination performance and reduce redundant variables, a genetic algorithm-based k-nearest neighbors (GA-KNN) [17] with a ten-fold cross-validation method was used for feature selection (repeats = 100). Ten well-known ML algorithms, including decision tree (DT), K-nearest neighbors (KNN), logistic regression (LR), naive Bayes (NB), random forest (RF), multiplayer perceptron (MLP), extreme gradient boosting machine (XGB), gradient boosting decision tree (GBDT), linear support vector machine (SVM-linear), and non-linear support vector machine (SVM-nonlinear), were selected to develop the CAS classification model. Their performance was assessed using both internal and external validation datasets. Given that LR algorithm is a highly interpretable and simplified ML algorithm, we used it as a performance reference.

To evaluate the model’s performance, we reported both the area under the receiver operating characteristic (auROC) curve and the precision-recall curve (auPRC). A calibration plot was used to assess the agreement between predictions and observations. The best cut-off point for each model was estimated using Youden's method, and the following metrics were also calculated to reflect the model performance: sensitivity, specificity, positive and negative predictive values (PPV and NPV), and positive and negative likelihood ratios (PLR and NLR).

CAS can be influenced by various factors. Among these, advanced age, obesity, history of hypertension, diabetes, and hyperlipidemia are significant risk factors for the development of CAS. To verify the stability of model performance, sensitivity analysis was used to explore the performance of the optimal model in five subgroups. Subgroup 1: individuals aged ≥ 65 years; subgroup 2: individuals whose BMI ≥ 30 kg/m^2^; subgroup 3: individuals with hypertension [18] (SBP ≥ 140 mmHg or DBP ≥ 90 mmHg); subgroup 4: individuals with diabetes [19] (FSG ≥ 7.0); and subgroup 5: individuals with dyslipidemia [20] (defined as TC ≥ 5.18 mmol/L or TG ≥ 1.76 mmol/L, or LDL-C ≥ 3.37 mmol/L, or HDL-C ≤ 1.04 mmol/L).

### Model interpretability and utility

To better understand the reasoning mechanism behind the high-performing ML model, we implemented the SHapley Additive exPlanations (SHAP) method using the SHAP package (https://github.com/slundberg/shap) for further analysis [21]. The clinical utility of each model was evaluated using Decision Curve Analysis (DCA).

### Statistical analysis

The dataset was cleaned up using the listwise method for excluding missing data and the Tukey method for identifying and eliminating outliers. For categorical variables, data were expressed as n (%) while continuous variables were expressed as mean ± SD, or for continuous variables with skewed distribution as median (interquartile range (IQR)). The chi-squared test, Students *T*-test, Mann–Whitney *U* test, or Kruskal–Wallis H test were all used to compare group differences based on variable distribution and comparison purpose. The models were developed with our own program built in Python (version 3.7; Python Software Foundation, Wilmington, DE, USA) using the scikit-learn package (version 0.24.0).

## Results

### Characteristics of the study populations

**Fig. 1:**
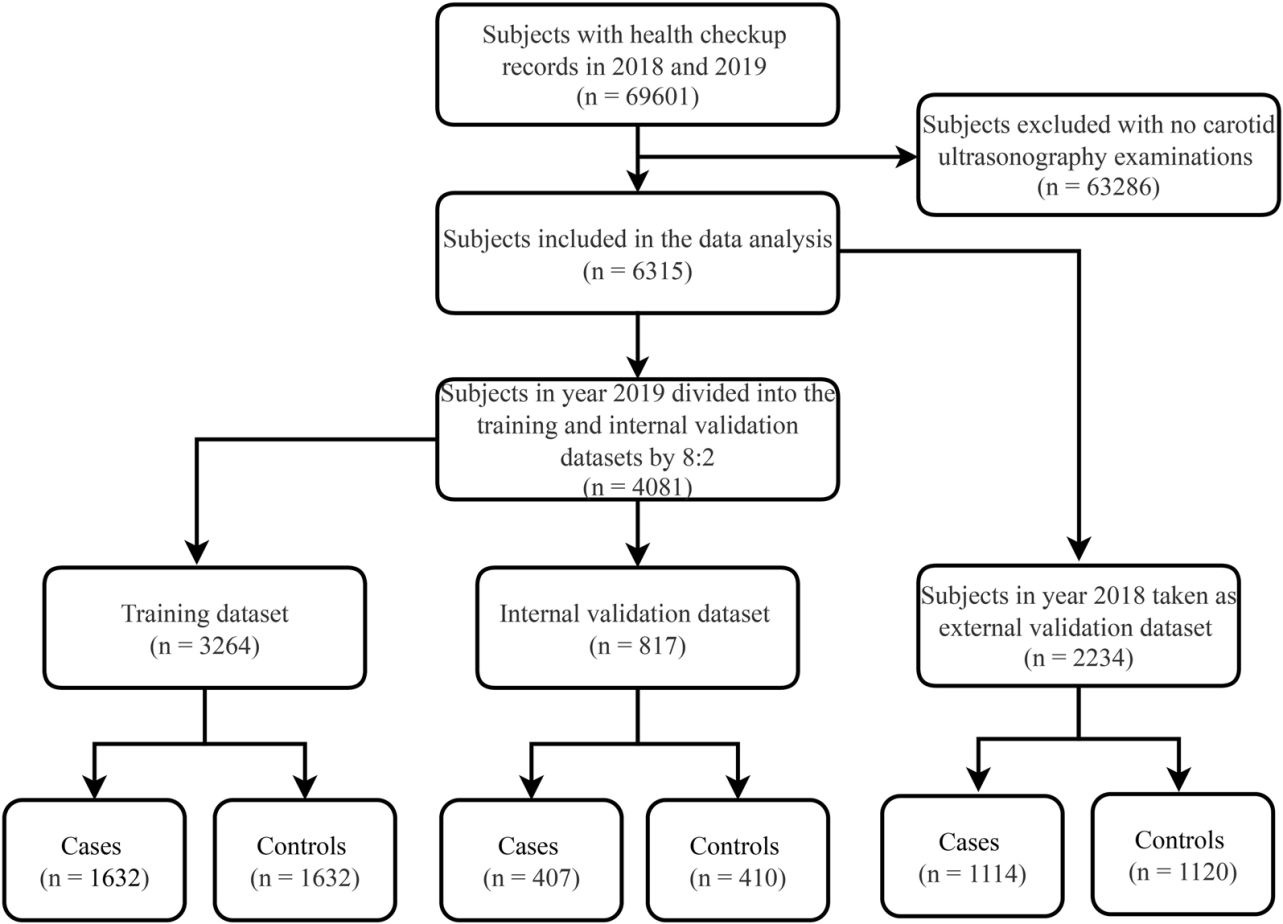
Flowchart of the study

A flowchart of the patient selection process is shown in Fig. 1. A total of 69,601 patients received health check-ups between 2018 and 2019. After excluding patients who did not undergo ultrasonography, data for 6315 patients were included in the analysis. Of these, 3264 CAS cases were included in the training dataset, 817 in the internal validation dataset, and 2234 in the external validation dataset. Table 1 presents the demographic and clinical characteristics of the training dataset, as well as internal and external validation datasets. The proportion of CAS in the training, internal, and external validation datasets was approximately 50%, and there were no statistically significant differences between the groups. When comparing the variables in each validation dataset with the training dataset, it was found that the AST was higher in the internal validation dataset than in the training dataset, with values of 20 (IQR: 17, 24) and 19 (IQR: 16, 24) U/L, respectively. In addition, age and waist circumference were higher in the external validation dataset than in the training dataset. The external validation dataset had an age of 49 (IQR: 40, 57) years old, while the training dataset had an age of 48 (IQR: 37, 55) years. Similarly, the external validation dataset had a waist circumference of 85 (IQR: 79, 91) cm, while the training dataset had a waist circumference of 85 (IQR: 78, 91) cm. However, HDL-C, TP, and ALB levels in the external validation set were found to be lower than levels in the training set. The HDL-C level in the external validation set was 1.20 (IQR: 1.01, 1.42) mmol/L, while in the training set it was 1.21 (IQR: 1.03, 1.46) mmol/L. The TP level was 69.1 (IQR: 66.8, 71.6) g/L in the external validation set and 70.2 (IQR: 67.8, 72.6) g/L in the training set. The ALB level was 43.80 (IQR: 42.30, 45.40) g/L in the external validation set and 44.30 (IQR: 42.70, 45.90) g/L in the training set. The other characteristics of each validation set were comparable to those of the training set.

**Table 1:** Characteristics of the training and validation datasets

Characteristics	Overall, N = 6315	Training set, N = 3264	Internal validation set (#), N = 817	External validation set (#), N = 2234
CAS proportion	3153 (50%)	1632 (50%)	407 (50%)	1114 (50%)
Age (years)	48 (38, 56)	48 (37, 55)	48 (37, 56)	49 (40, 57)***
Sex (n,%)				
Female	4196 (66%)	2137 (65%)	550 (67%)	1509 (68%)
Male	2119 (34%)	1127 (35%)	267 (33%)	725 (32%)
BMI (kg/m^2^)	25.4 (23.2, 27.7)	25.4 (23.1, 27.7)	25.6 (23.3, 27.7)	25.4 (23.3, 27.8)
Waist circumference (cm)	85 (79, 91)	85 (78, 91)	85 (78, 91)	85 (79, 91)*
Height (cm)	169 (162, 175)	169 (162, 175)	170 (163, 175)	169 (163, 174)
Body weight (kg)	73 (63, 82)	72 (63, 82)	73 (64, 82)	73 (63, 82)
SBP (mmHg)	126 (115, 140)	126 (115, 140)	126 (115, 141)	126 (115, 141)
DBP (mmHg)	76 (68, 85)	76 (68, 85)	76 (67, 84)	76 (68, 85)
FPG (mmol/L)	5.18 (4.85, 5.61)	5.17 (4.84, 5.61)	5.16 (4.84, 5.59)	5.19 (4.87, 5.61)
TG (mmol/L)	1.38 (0.93, 2.10)	1.37 (0.92, 2.06)	1.38 (0.91, 2.18)	1.39 (0.94, 2.12)
TC (mmol/L)	4.87 (4.32, 5.49)	4.90 (4.33, 5.51)	4.84 (4.27, 5.48)	4.84 (4.32, 5.48)
HDL-C (mmol/L)	1.20 (1.02, 1.44)	1.21 (1.03, 1.46)	1.21 (1.01, 1.46)	1.20 (1.01, 1.42)**
LDL-C (mmol/L)	3.08 (2.58, 3.61)	3.09 (2.58, 3.62)	3.06 (2.50, 3.61)	3.09 (2.59, 3.61)
Non-HDL-C (mmol/L)	3.63 (3.04, 4.25)	3.65 (3.04, 4.28)	3.59 (3.00, 4.21)	3.62 (3.04, 4.21)
ALP (U/L)	65 (55, 77)	65 (54, 77)	66 (55, 78)	65 (55, 78)
GGT (U/L)	25 (17, 40)	25 (16, 39)	26 (17, 40)	25 (17, 40)
ALT (U/L)	20 (14, 30)	20 (14, 30)	21 (15, 31)	20 (14, 29)
AST (U/L)	20 (16, 24)	19 (16, 24)	20 (17, 24)*	20 (17, 24)
TP (g/L)	69.8 (67.4, 72.3)	70.2 (67.8, 72.6)	70.2 (67.6, 72.6)	69.1 (66.8, 71.6)***
ALB (g/L)	44.10 (42.60, 45.70)	44.30 (42.70, 45.90)	44.30 (42.70, 45.70)	43.80 (42.30, 45.40)***
TBIL (umol/L)	12.8 (10.0, 16.4)	12.7 (10.0, 16.3)	13.2 (10.1, 16.5)	12.8 (10.0, 16.4)
BUN (mmol/L)	5.07 (4.32, 5.92)	5.08 (4.31, 5.90)	5.05 (4.35, 5.88)	5.04 (4.31, 5.97)
Cr (μmol/L)	67 (56, 77)	67 (56, 77)	68 (56, 78)	68 (57, 77)
UA (μmol/L)	349 (285, 412)	348 (284, 409)	347 (281, 411)	350 (286, 417)

Characteristics are presented as median (interquartile range) for continuous features and frequencies (%) for categorical features

*ALB*  albumin; *ALP* alkaline phosphatase; *ALT* alanine aminotransferase; *AST* aspartate aminotransferase; *BMI* body mass index; *BUN* blood urea nitrogen; *CAS* carotid atherosclerosis; *Cr* creatine; *DBP* diastolic blood pressure; *FPG* fasting plasma glucose; *GGT* gamma-glutamyl transpeptidase; *HDL-C* high-density lipoprotein-C; *LDL-C* low-density lipoprotein-C; *non-HDL-C * non high-density lipoprotein cholesterol; *SBP* systolic blood pressure; *TC* total cholesterol; *TG* triglyceride; *TP* total protein; *TBIL* total bilirubin; *UA* uric acid

^#^Comparing each validation set to the training set

^*^P-value < 0.05; **P-value < 0.01; ***P-value < 0.001

### Development and calibration of CAS classification ML models

**Fig. 2:**
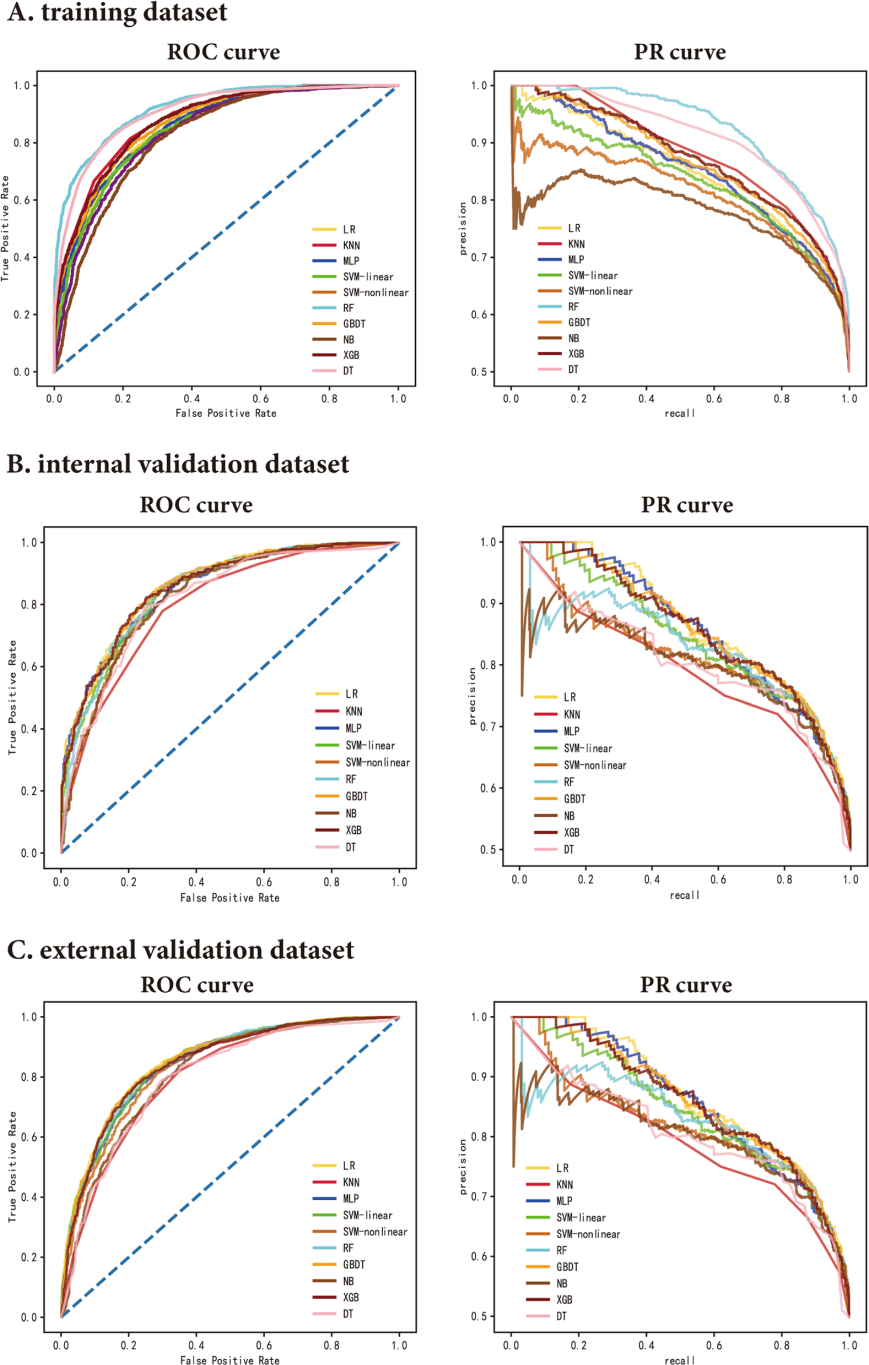
ROC and PR curves of models with different algorithms in the training, internal validation, and external validation datasets. PR: precision-recall; ROC: receiver operating characteristic; KNN k-nearest neighbors; LR logistic regression; NB naive bayes; RF random forest; SVM-linear linear support vector machine; SVM-nonlinear non-linear support vector machine; DT decision tree; GBDT gradient boosting decision tree; MLP multiplayer perception; XGB extreme gradient boosting machine

**Fig. 3:**
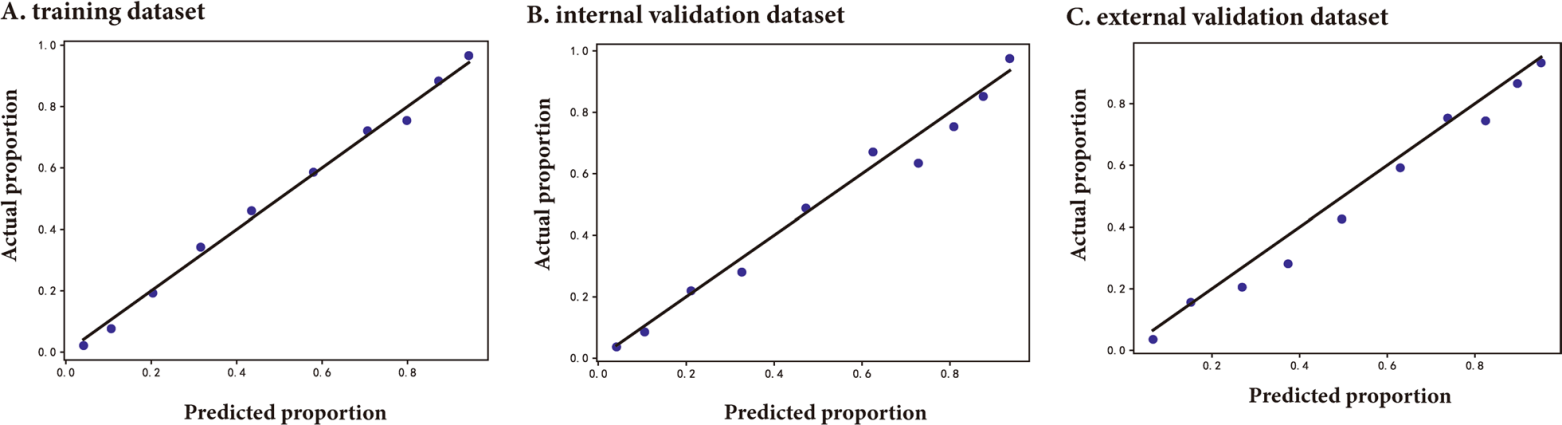
Calibration plots of the GBDT model in the training, internal validation, and external validation datasets. GBDT: gradient boosting decision tree

Ten features for CAS classification were selected using the GA-KNN algorithm from 24 candidate variables, including age, sex, non-HDL-C, FSG, TC, DBP, LDL-C, ALB, GGT, and ALP. Table 2 and Fig. 2 provide a summary of the performance of ML models. In the internal validation set, LR and GBDT models had the best performance with an auROC up to 0.861 (95% CI 0.841–0.881) and 0.860 (95% CI 0.839–0.880), whereas the corresponding performance metrics of KNN, MLP, SVM-linear, SVM-nonlinear, RF, NB, XGB, and DT were 0.800 (95% CI 0.777–0.824), 0.852 (95% CI 0.832–0.872), 0.846 (95% CI 0.824–0.867), 0.835 (95% CI 0.812–0.857), 0.849 (95% CI 0.828–0.870), 0.829 (95% CI 0.805–0.852), 0.855 (95% CI 0.835–0.876), and 0.817 (95% CI 0.794–0.840), respectively. The model performance reflected by auPR is consistent with that of auROC. With a cut-off value of the operating point determined by the maximal Youden index, the specificity, sensitivity, PPV, NPV, PLR, and NLR were 0.85, 0.722, 0.757, 0.804, 3.057, and 0.208 for the LR model, respectively, and 0.84, 0.729, 0.762, 0.797, 3.104, and 0.219 for the GBDT model, respectively. External validation was also performed to validate the model performance of CAS discrimination, and LR and GBDT models demonstrated similar performance in auROC, auPR, sensitivity, and specificity (Fig. 2). We also showed the calibration curves for the GBDT model in the training, internal and external validation dataset in Fig. 3, which showed good consistency between actual and expected probabilities.

**Table 2:** The performance of ten ML models for recognizing CAS in the training set, internal validation set, and external validation set

Datasets	Model performance	LR	KNN	MLP	SVM-linear	SVM-nonlinear	RF	GBDT	NB	XGB	DT
Training set	auROC (95% CI)	0.855 (0.845–0.865)	0.878 (0.868–0.888)	0.856 (0.845–0.866)	0.847 (0.836–0.858)	0.837 (0.825–0.848)	0.921 (0.914–0.928)	0.868 (0.858–0.878)	0.822 (0.811–0.834)	0.875 (0.865–0.884)	0.908 (0.900–0.916)
	auPR (95% CI)	0.849 (0.835–0.862)	0.852 (0.838–0.865)	0.851 (0.837–0.864)	0.833 (0.818–0.849)	0.812 (0.794–0.829)	0.925 (0.917–0.933)	0.865 (0.852–0.877)	0.777 (0.757–0.798)	0.871(0.859–0.883)	0.903 (0.892–0.913)
	Threshold^a^	0.547	0.594	0.517	0.539	0.523	0.662	0.57	0.51	0.702	0.652
	Sensitivity	0.77	0.814	0.792	0.82	0.814	0.831	0.806	0.801	0.858	0.841
	Specificity	0.776	0.779	0.725	0.719	0.708	0.831	0.764	0.709	0.844	0.811
	PPV	0.766	0.787	0.744	0.745	0.762	0.813	0.782	0.766	0.833	0.817
	NPV	0.776	0.808	0.773	0.798	0.757	0.848	0.782	0.718	0.865	0.836
	PLR	3.444	3.692	2.878	2.921	2.792	4.931	3.416	2.754	5.49	4.455
	NLR	0.296	0.238	0.287	0.25	0.262	0.203	0.254	0.28	0.168	0.196
Internal Validation set	auROC (95% CI)	0.861(0.841–0.881)	0.800(0.777–0.824)	0.852(0.832–0.872)	0.846(0.824–0.867)	0.835(0.812–0.857)	0.849(0.828–0.870)	0.860(0.839–0.880)	0.829(0.805–0.852)	0.855(0.835–0.876)	0.817(0.794–0.840)
	auPR (95% CI)	0.864(0.842–0.885)	0.757(0.725–0.791)	0.857(0.834–0.880)	0.842(0.816–0.867)	0.816(0.785–0.847)	0.826(0.795–0.861)	0.860(0.836–0.883)	0.799(0.766–0.834)	0.857(0.834–0.880)	0.790(0.759–0.823)
	Sensitivity	0.85	0.779	0.855	0.867	0.855	0.838	0.84	0.838	0.811	0.789
	Specificity	0.722	0.7	0.646	0.693	0.7	0.727	0.729	0.685	0.746	0.749
	PPV	0.757	0.72	0.724	0.74	0.759	0.751	0.762	0.769	0.753	0.738
	NPV	0.804	0.761	0.759	0.83	0.782	0.812	0.797	0.73	0.797	0.78
	PLR	3.057	2.596	2.418	2.822	2.85	3.067	3.104	2.663	3.196	3.139
	NLR	0.208	0.316	0.224	0.192	0.207	0.223	0.219	0.237	0.253	0.282
External Validation set	auROC (95% CI)	0.853 (0.839–0.866)	0.800 (0.783–0.815)	0.841 (0.827–0.854)	0.847 (0.833–0.859)	0.833 (0.818–0.846)	0.847 (0.833–0.860)	0.851 (0.837–0.863)	0.812 (0.796–0.826)	0.844 (0.829–0.856)	0.799 (0.783–0.814)
	auPR (95% CI)	0.844 (0.824–0.860)	0.750 (0.726–0.772)	0.828 (0.808–0.846)	0.836 (0.816–0.854)	0.816 (0.794–0.836)	0.829 (0.809–0.848)	0.835 (0.813–0.853)	0.767 (0.742–0.793)	0.824 (0.801–0.843)	0.761 (0.738–0.785)
	Sensitivity	0.782	0.82	0.811	0.81	0.811	0.799	0.794	0.838	0.81	0.781
	Specificity	0.781	0.651	0.695	0.735	0.719	0.756	0.765	0.652	0.728	0.713
	PPV	0.748	0.7	0.71	0.722	0.741	0.731	0.75	0.736	0.728	0.718
	NPV	0.813	0.784	0.798	0.827	0.787	0.805	0.803	0.725	0.808	0.775
	PLR	3.574	2.348	2.658	3.053	2.882	3.278	3.383	2.405	2.973	2.716
	NLR	0.279	0.277	0.271	0.259	0.264	0.266	0.269	0.249	0.262	0.307

*auROC* area under the receiver operating characteristic curve; *auPR* area under the Precision-Recall curve; *KNN* k-nearest neighbors; *LR* logistic regression; *NB* naive bayes; *RF* random forest; *SVM-linear* linear support vector machine; *SVM-nonlinear* non-linear support vector machine; *DT* decision tree; *GBDT* gradient boosting decision tree; *MLP* multiplayer perception; *XGB* extreme gradient boosting machine; PPV positive predictive value; *NPV* negative predictive value; *PLR* positive likelihood ratio; *NLR* negative likelihood ratio

^a^Calculated at the operating point determined by the Youden Index

### Sensitivity analysis of the optimal GBDT model for CAS classification

To test performance of the GBDT model in different CAS risk groups, sensitivity analysis was performed in the following five subsets, individuals aged ≥ 65 years, BMI ≥ 30 kg/m^2^, with dyslipidemia, with hypertension, or with diabetes in the training and internal and external validation datasets (Table 3). The analysis showed moderate to high discriminative performance of GBDT models across different subgroups, with an auROC range of 0.869–0.996, auPR of 0.866–0.993, sensitivity of 0.710–0.948, specificity of 0.775–0.939, PPV of 0.645–0.951, PLR of 0.93–3.184 and NLR of 0.133–0.689. However, the NPV was relatively low in subgroup aged ≥ 65 years, with a range of 0.100–0.333 and in subgroup with diabetes with a range of 0.361–0.548.

**Table 3:** Performance of GBDT model in five high-risk CAS subgroups

Disease subgroups	Datasets	auROC (95% CI)	auPR (95% CI)	Sensitivity	Specificity	PPV	NPV	PLR	NLR
Age ≥ 65	Training set (N = 239)	0.996(0.989–1)	NA	0.941	1.000	0.930	0.100	1.019	0.689
	Internal validation set (N = 64)	NA	NA	1.000	1.000	0.951	0.333	1.289	0.133
	External validation set (N = 252)	NA	NA	1.000	1.000	0.943	0.200	1.053	0.253
BMI ≥ 30	Training set (N = 341)	0.927(0.904–0.949)	0.939(0.917–0.96)	0.824	0.874	0.708	0.691	2.122	0.390
	Internal validation set (N = 68)	NA	NA	1.000	1.000	0.645	0.676	2.045	0.540
	External validation set (N = 213)	0.971(0.954–0.984)	0.972(0.954–0.985)	0.906	0.925	0.714	0.664	2.524	0.511
Dyslipidemia	Training set (N = 3027)	0.869(0.858–0.879)	0.866(0.852–0.878)	0.797	0.784	0.751	0.756	3.014	0.321
	Internal validation set (N = 754)	0.922(0.907–0.937)	0.928(0.911–0.943)	0.894	0.788	0.743	0.759	2.890	0.318
	External validation set (N = 2070)	0.877(0.864–0.888)	0.877(0.861–0.891)	0.822	0.775	0.761	0.773	3.184	0.293
Hypertension	Training set (N = 897)	0.87(0.85–0.89)	0.945(0.933–0.957)	0.710	0.855	0.793	0.611	1.474	0.244
	Internal validation set (N = 235)	0.978(0.962–0.99)	0.992(0.985–0.997)	0.948	0.903	0.802	0.491	1.453	0.372
	External validation set (N = 658)	0.895(0.875–0.916)	0.951(0.939–0.963)	0.804	0.829	0.789	0.667	1.621	0.217
Diabetes	Training set (N = 241)	0.973(0.954–0.989)	0.993(0.986–0.997)	0.911	0.939	0.824	0.361	1.198	0.452
	Internal validation set (N = 49)	NA	NA	1.000	1.000	0.870	0.000	0.930	NA
	External validation set (N = 128)	NA	NA	1.000	1.000	0.825	0.548	1.702	0.298

*GBDT* gradient boosting decision tree; *auROC* area under the receiver operating characteristic curve; *auPR* area under the Precision-Recall curve; *PPV* positive predictive value; *NPV* negative predictive value; *PLR* positive likelihood ratio; *NLR* negative likelihood ratio; *NA* not applicable

### Interpretability and clinical benefit analysis

**Fig. 4:**
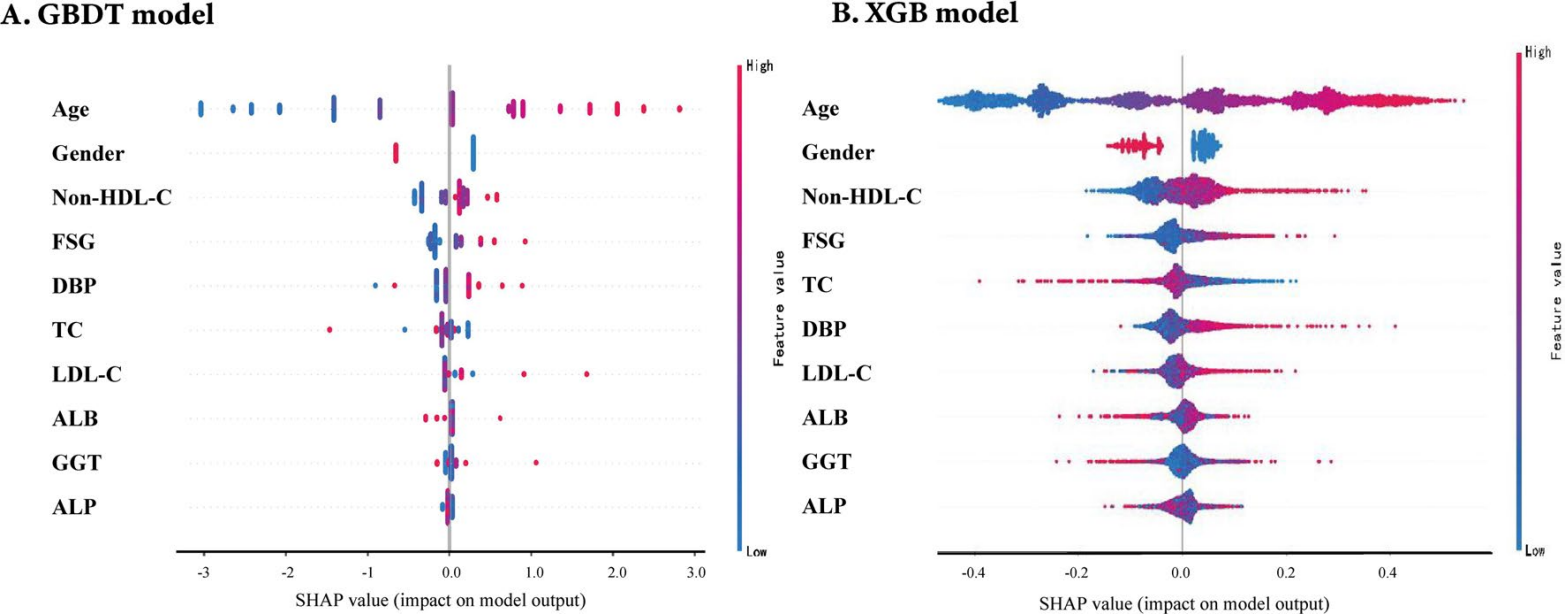
Contribution analysis to the prediction of the GBDT and XGB models in the training dataset using the SHAP technique. The higher the ranking, the more important the characteristics; each point is a patient and the color gradient from red to blue corresponds to the high- to low-value of this feature. The point on the left side of the digital baseline (with a SHAP value of 0) represents a negative contribution to suffering from CAS, while the point on the right represents a positive contribution. The farther from the baseline, the greater the impact. CAS: carotid atherosclerosis; GBDT: gradient boosting decision tree; SHAP: SHapley Additive exPlanations; XGB: extreme gradient boosting machine; ALB Albumin; ALP Alkaline phosphatase; DBP Diastolic blood pressure; FSG Fasting serum glucose; GGT Gammaglutamyl transpeptidase; LDL-C Low-density lipoprotein cholesterol; Non-HDL-C Non-high-density lipoprotein cholesterol; TC Total cholesterol

**Fig. 5:**
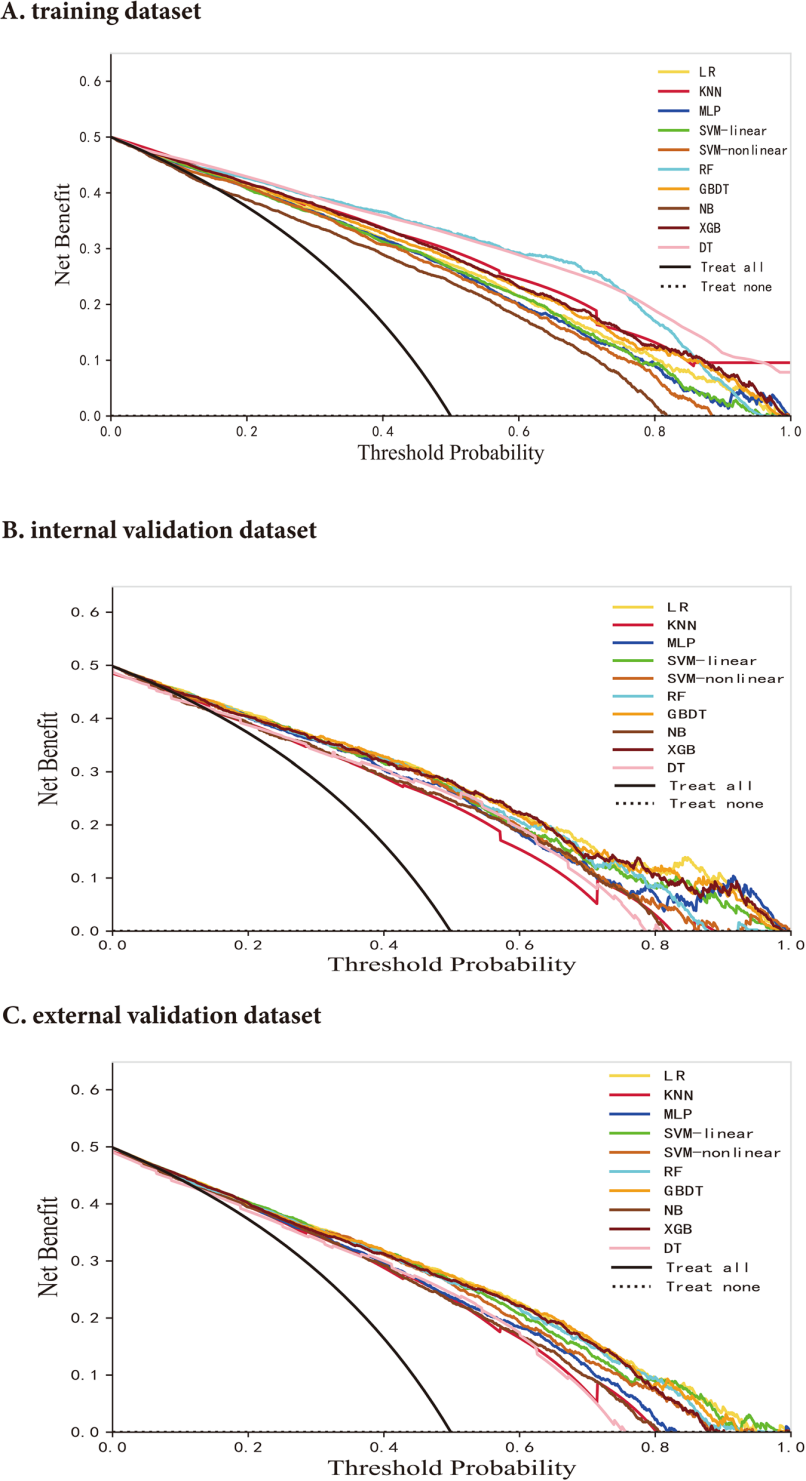
DCA curve analysis of the ML models in the development and validation datasets. DCA: decision curve analysis; KNN k-nearest neighbors; LR logistic regression; NB naive bayes; RF random forest; SVM-linear linear support vector machine; SVM-nonlinear non-linear support vector machine; DT decision tree; GBDT gradient boosting decision tree; MLP multiplayer perception; XGB extreme gradient boosting machine

Finally, the GBDT model with the best performance was selected for SHAP analysis. We also performed SHAP analysis on the XGB model, which is an integrated learning algorithm based on GBDT. Figure 4 shows a global summary of the SHAP value distribution for all features, which helps to understand the importance of each feature. Age, gender, Non-HDL-C, FSG, DBP, and TC were identified as the top six influencing indicators for CAS classification. According to both the GBDT and XGB models, age, Non-HDL-C, FSG, and DBP showed a positive correlation with CAS, while gender and TC showed a negative correlation with CAS (Figs. 4a, b). Age is the factor that contributes the most to model predictions. The clinical utility of ML models at varying risk thresholds is depicted in Fig. 5. The ML models demonstrated a net benefit in DCA when compared to “treat-all”, “treat-none” at a threshold probability of  > 20%. Here, “treat” refers to the selection of patients for intervention.

## Discussion

This study developed and validated a screening model for CAS using ten ML algorithms based on routine clinical and laboratory features. The results showed that the GBDT models provided the best discriminatory performance (maximum auROC and auPR in validation datasets). At the same time, other metrics outperformed other ML models in both internal and external validation sets, demonstrating the utility of the best model. We further performed an interpreted analysis of the model and found that age was the most critical factor for the GBDT model for decision-making. Other important factors included sex, non-HDL-C and SBP. Compared to previous studies, this study has the following advantages. First, we used the GA-KNN algorithm to select the optimal combination of features. Second, the model was validated using an external dataset, which further confirmed its ability to discriminate. Third, the SHAP algorithm compensates for the “black box” problem of advanced ML algorithms [21]. This study can be seen as the first step in the use of ML models for screening of CAS in clinical practice, and can serve as a reference for furtherresearch in the future.

GA-KNN algorithm was used to select the optimal combination of candidate variables for CAS classification in our study. Compared to Shao’s study. [11], which modeled carotid plaque classification in physical examination populations, five same predictive variables for model construction (age, sex, blood pressure, glucose, and serum lipids) were used. Similar findings support the reliability of the GA-KNN feature selection algorithm. In terms of the number of selected indicators included in the model, Fan et al. used 19 features from different medical examination packages [13], with the possibility of collinearity, which may bias the model predictions. In addition, two other studies selected nonclinical indicators, such as nonalcoholic fatty liver disease and homocysteine in Yu et al. [12] and platelets and diabetes mellitus in Fan et al. [13]. The inclusion of these uncommon indicators greatly limits the scope of application of the model. Our study used a genetic algorithm combined with the KNN algorithm to find the optimal feature combination of routine health check-up indicators. This approach helps to avoid the underlying bias caused by a lack of experience in manually selecting features. This technique is worthy of further validation and evaluation in future studies [23].

Our study found that GBDT algorithms achieved the best performance in CAS classification, which is significantly better than that of other reported ML models [23]. The reasons for the better model performance of GBDT in our study may be explained as follows: First, we used a feature selection strategy to find the best combination of CAS predictors to ensure that the selection retains important information and avoids information redundancy. Second, although the superiority of LR as a classical linear statistical analysis model was confirmed in a previous study [13], the GBDT model in our study used different computational strategies and also achieved similar performance. In terms of algorithm principle, GBDT is a classical tree-integrated boosting algorithm, which can identify non-linear and interconnected correlations between input and output [24]. It is also worth mentioning that although the XGB algorithm is modified from GBDT, the XGB model in our study does not perform as well as the GBDT model. The underlying reason may be the XGB model with more parameters and tuning, and prone to overfitting than GBDT for real-world EHR data. Therefore, the GBDT algorithm can be considered a powerful tool for analyzing real-world EHR data.

In addition, subgroup analysis showed that the performance of the established GBDT models had a low NPV in the subgroup aged ≥ 65 years or with diabetes, indicating that this model were not specific enough to exclude patients with low CAS risk among the above two subgroups. The underlying reasons may be the small number of negative samples and high prevalence of CAS in this subgroup, which led to insufficient training of the model's discrimination ability for CAS negative individuals. Another reason may be that the features selected in our study did not have adequate diagnostic capabilities for seniors and diabetes patients, suggesting that adding specific predictors with the most discriminatory power (e.g., risk genes) improves model performance in the future. In addition, patients in both subgroups may frequently have several underlying diseases, which may have an impact on the model’s discrimination power. Finally, before ML modeling, we can consider conducting cluster analysis [25] to explore the heterogeneity of the target population, so as to guide the construction of the ML model and achieve a balanced performance between bias and variance.

Considering the “black box” nature of the advanced ML model, this study also used the SHAP algorithm, which can be applied to any type of ML model, has the advantages of fast implementation of tree-based models, and can ensure consistency and local accuracy, to conduct interpretability analysis of GBDT and XGB models. For the first time, we ranked the factors affecting CAS, and found that age and sex were the first two key factors for GBDT models in CAS classification. The potential mechanism may be corroborated by previous findings that age and sex may influence CAS distribution and ultrasound morphology [26, 27], and indicated that age and sex differences should be considered in clinical practice [28]. In addition, consistent with previous findings, Non-HDL-C, FSG, and SBP were also important predictors for CAS classification [29], suggesting that CAS is a metabolically closely related disease [27] and that above metabolic indicators should be paid more attention for CAS prevention [3].

There were several limitations in our study: (1) Although we used internal and external validation datasets to assess the model's application stability, the risk and benefit of the optimal model deployed in real-world scenarios requires the design of clinical trials for further evaluation. (2) Information on medications could not be collected from health check-up records. However, preparation prior to the physical examination, including monitor diet (e.g. not eating too much greasy and indigestible food), not taking non-essential medicines three days before the physical examination, and not drinking water or eating food on the day of the physical examination, can minimize the impact of potential interfering factors. (3) As this study was based on physical examination data from people in Northeast China, the reliability of the established models needs further validation if they are to be applied to scenarios beyond the population representation in this study.

## Conclusions

The ML models developed could provide good power for CAS identification, which will hopefully be applied in scenarios without ethnic and geographic heterogeneity, and guide prevention and management of individuals at risk of CAS.

## Data Availability

The corresponding author can provide all the data sets used in this work upon reasonable request.

## Abbreviations

ALB: Albumin
ALP: Alkaline phosphatase
ALT: Alanine transaminase
AST: Aspartate aminotransferase
auPR: Area under the precision-recall curve
auROC: Area under the receiver operating characteristic curve
BMI: Body mass index
BUN: Blood urea nitrogen
CAS: Carotid atherosclerosis
Cr: Creatinine
DBP: Diastolic blood pressure
DCA: Decision curve analysis
DT: Decision tree
FSG: Fasting serum glucose
GA-KNN: Genetic algorithm based k-nearest neighbors
GBDT: Gradient boosting decision tree
GGT: Gammaglutamyl transpeptidase
HDL-C: High-density lipoprotein cholesterol
IMT: Carotid intima-media thickness
IQR: Interquartile range
KNN: K-nearest neighbor
LDL-C: Low-density lipoprotein cholesterol
LR: Logistic regression
ML: Machine learning
MLP: Multiplayer perception
NB: Naive bayes
NLR: Negative likelihood ratio
Non-HDL-C: Non-high-density lipoprotein cholesterol
NPV: Negative predictive value
PLR: Positive likelihood ratio
PPV: Positive predictive value
RF: Random forest
SBP: Systolic blood pressure
SD: Standard deviation
SHAP: Shappley additive explanations
SVM-linear: Linear support vector machine
SVM-nonlinear: Non-linear support vector machine
TBIL: Total bilirubin
TC: Total cholesterol
TG: Triglyceride
TP: Total protein
UA: Uric acid
XGB: Extreme gradient boosting machine
